# Consensus on the investigation of thrombophilia in women and clinical management

**DOI:** 10.31744/einstein_journal/2019AE4510

**Published:** 2019-08-13

**Authors:** Claudia Mac Donald Bley Nascimento, Andréa Maria Novaes Machado, João Carlos de Campos Guerra, Eduardo Zlotnik, Dirceu Hamilton Cordeiro Campêlo, Paulo Kauffman, Hilton Waksman, Nelson Wolosker, Sérgio Podgaec, Nelson Hamerschlak

**Affiliations:** 1 Hospital Israelita Albert Einstein, São Paulo, SP, Brazil.; 2 Hospital das Clínicas, Faculdade de Medicina, Universidade de São Paulo, São Paulo, SP, Brazil.

**Keywords:** Thrombophilia, Antiphospholipid syndrome, Venous thromboembolism, Pregnancy, Abortion, habitual, Pregnancy complications, Contraception, Venous thrombosis

## Abstract

**Objective:**

To standardize the investigation and clinical management of women with laboratory and/or clinical abnormalities suggestive of thrombophilia, in order to optimize antithrombotic approach and indication of laboratory tests.

**Methodology:**

A discussion was carried out among 107 physicians (gynecologists/obstetricians, hematologists and vascular surgeons) present at a forum held at the *Hospital Israelita Albert Einstein*, in São Paulo (SP), Brazil. As a minimum criterion, 80% agreement was established in the voting to each recommendation of conduct in the final document. The cases in which there was agreement below 80% were discussed again, reaching a consensual agreement of conduct for the document writing.

**Conclusion:**

The standardization of an institutional consensus of suggestions of clinical approach contributes to a better management of the group to be evaluated and minimizes risks of intercurrent events. This was the first national consensus on the investigation of thrombophilia in women.

## INTRODUCTION

Thrombophilia is defined as a tendency towards thrombosis resulting from hereditary alterations (deficiency of proteins C, S and antithrombin; factor V Leiden − FVL − mutation; and protrombin gene mutation) or acquired from coagulation (antiphospholipid syndrome) that lead to a state of pro-thrombosis, which predisposes people to present with venous or arterial thrombosis. The etiology of thrombosis (arterial or venous occlusion of the blood vessel by a clot) is multifactorial, and the presence of a genetic or acquired laboratory modification of thrombophilia is only one of the many factors that determine its risk, as it has little prevalence in the general population, as is shown in table 1. Obesity, use of hormones, surgery, and long periods in bed, long flights, cancer, smoking, and pregnancy are factors that increase the thrombotic risk in an independent manner.^(1)^

**Table 1:** Prevalence of thrombophilia and estimated relative risk for various clinical manifestations(1)

	Deficiency of antithrombin	Deficiency of protein C	Deficiency of protein S	Factor V Leiden	Mutation of the protrombin gene (20210A)
Prevalence in the general population, %	0.02	0.2	0.03-0.13	3.0-7.0	0.7-4.0
Prevalence in patients with recurring VTE, %	1.0	3.0	2.0	20.0	5.0
Relative risk for the first manifestation of VTE	5-10	4-6.5	1-10	3-5	2-3
Relative risk for recurrence of VTE	1.9-2.6	1.4-1.8	1.0-1.4	1.4	1.4
Relative risk for arterial thrombosis	Without substitution	Without consistent substitution	Without consistent substitution	1.3	0.9
Relative risk for obstetric complications	1.3-3.6	1.3-3.6	1.3-3.6	1.0-2.6	0.9-1.3

VTE: venous thromboembolism.

Pregnancy is associated with physiological changes that affect coagulation and the fibrinolytic system. An imbalance in this system leads to a state of hypercoagulability, and pregnant women have, therefore, an increased risk of venous thromboembolism (VTE) events.^(2)^ The incidence of thromboembolism in pregnant women is 0.6 to 1.7 case in 1,000 gestations.^(3)^

Approximately 50 to 60% of these thrombosis cases occur during the puerperium (up to 6 weeks postpartum).^(4)^

Despite the risk of VTE being about four times greater in pregnant than in non-pregnant women at child-bearing age,^(5)^ there is no scientific evidence that including thrombophilia investigation during the prenatal care period, as is often requested, is useful.

Literature recommends that the laboratory investigation of thrombophilia be guided by the history of the patient, family history of thrombosis, and suggested when its result will partially affect the clinical management. It should be used in the following situations: ^(1,6-8)^

- Patients with a history of thrombosis and their first-degree kinship, if the knowledge of the laboratory test results modifies clinical management.

- Patients with a history of repeated late-term miscarriage or early miscarriages.

Cost-effectiveness studies have shown that the indiscriminate use of thrombophilia tests in the general population were not effective in preventing deaths or other events.^(9-11)^

## OBJECTIVE

With the purpose of standardizing the laboratory investigation of thrombophilia in the female population, we decided, along with the medical staff of *Hospital Israelita Albert Einstein*, to create the Thrombophilia Forum with hematologists, vascular surgeons, and gynecologists/obstetricians of the organization, to evaluate the true importance of this investigation, its management, and to define, by consensus, the standardization of measures that should be taken in caring for our target population.

The Thrombophilia Forum was held on June 10^th^, 2017, at *Hospital Israelita Albert Einstein*, in the city of São Paulo (SP), Brazil.

## METHODOLOGY

The hematology team of *Hospital Israelita Albert Einstein* (HIAE) performed a search, in April 2017, of scientific articles published in PubMed^®^/MEDLINE^®^ between January 2007 and January 2017, inserting the words in English: “thrombophilia”, “thrombosis and pregnancy”, “thrombophilia and screening”, “pre-hormone therapy”. The retrieved articles were analyzed as to their relevance, scope, and study design, which generated a list of randomized clinical studies, meta-analyses, systematic reviews, and guidelines,^(1,6-11)^ which was forwarded as a suggestion of reading via e-mail to the medical staff of the 539 specialist physicians registered in hematology, vascular surgery, and gynecology and obstetrics. It was accompanied by an invitation letter to a forum scheduled 2 weeks after the receipt of the e-mail. Of these, 107 physicians (80% gynecologists/obstetricians, 10% hematologists, and 10% vascular surgeons) attended the Forum that lasted 4 hours and 30 minutes and was composed of three stages: theoretical class to expose the theme of thrombophilia in women; interactive presentation of clinical cases in which the participants of the forum could expose their questions and comments; and exposure of a questionnaire prepared by the hematology team of HIAE, with 21 statements that illustrated the main medical managements about the topic addressed (thrombophilia in women). The participants should assess each statement and vote as true, to agree with the medical management explained, or false, to disagree with it. An electronic voting system was installed in the auditorium, with individual equipment, allowing the recording of the percentage of answers (true and false).

A 80%-agreement in the vote for insertion of each recommendation was established by the group of physicians present at the Forum as a minimal criterion of acceptable resolution, so that the statement was inserted into the final document. Cases in which there was agreement lower than 80% were again discussed, and a consensual agreement was reached for majority conduct.

## RECOMMENDATIONS

After literature review, reading of the bibliography, exposition of the literature in a plenary meeting, dynamic discussion of clinical cases, and voting on the consensus, we reached agreement of more than 80% in 95% of questions (20 out of 21), whereas only one of the questions had less than 80% votes. This question, after ample discussion, was reformulated and agreed upon by unanimity.

The guidelines were summarized on table 2.

**Table 2:** General recommendations and percentage of agreement of this consensus on thrombophilia

	General recommendations	Agreement (%)
**Screening**	1. Investigate thrombophilia in women with a history of VTE	85
	2. Investigate thrombophilia in first-degree kinship family members of patients with a history of VTE and who can potentially be exposed to thrombogenic factors	85
	3. Investigate thrombophilia in women with repeated miscarriages	88
	4. Routine research of thrombophilia in all pregnant women in prenatal care is not recommended	99
	5. It is not recommended to perform pre-contraception or pre-hormone replacement screening in the female population	96
	6. Perform screening of pre-hormonal use in women with history of thrombosis or with a positive family history (first-degree kinship)	100
**Time of the investigation**	7. Avoid performing laboratory investigation for VTE during the acute phase of thrombosis, since a reduction in plasma levels of protein C, S, and AT may occur	90
	8. The best time to perform laboratory investigation of VTE is at the end of anticoagulation, as of 2 to 4 weeks after anticoagulation is discontinued	94
	9. Genetic tests for FVL and investigation of the mutation of prothrombin, anti-cardiolipin, and anti-beta2-glycoprotein I do not suffer interference during the acute phase of thrombosis	88
**Use of hormones**	10. Women with a prior history of thrombosis related to hormones present with a contraindication for the use of hormones, except for progesterone IUD	95
	11. Women with laboratory modification in thrombophilia and with no history of thrombosis can use hormones with extreme care	86
	12. The use of a levonorgestrel IUD is safe in patients with thrombophilia	100
**Pregnancy and puerperium**	13. Women with a prior VTE event associated with a transient risk and not related to pregnancy/contraception should maintain vigilance during gestation and during puerperium; they should perform antithrombotic prophylaxis for 6 weeks	95
	14. Women with a previous VTE event associated with pregnancy/contraception should perform antithrombotic prophylaxis during a gestation and puerperium	98
	15. Women with FVL thrombophilia in homozygosis or mutant prothrombin in homozygosis, with no previous VTE event and no family history of thrombosis, should perform prophylaxis during pregnancy and puerperium	100*
	16. Women with FVL thrombophilia in homozygosis or mutant prothrombin in homozygosis, with no previous VTE event and with a family history of thrombosis should perform antithrombotic prophylaxis only in the postpartum period	98
	17. Women with thrombophilia (all other thrombophilias, except FVL homozygous and mutation of prothrombin 20210 homozygous), with no previous thromboembolism and with no family history of thrombosis should perform clinical vigilance before the delivery, and in the puerperium	92
	18. Women with thrombophilia (all other thrombophilias, except FVL homozygous and prothrombin mutation 20210), with no previous thromboembolism and with a family history of thrombosis should be on clinical surveillance during pregnancy and antithrombotic prophylaxis in the puerperium	94
	19. Women with antiphospholipid syndrome should use antithrombotic prophylaxis both during the antepartum period and the puerperium, combined with a low dose of aspirin (75-100mg/day)^†^	98
	20. In pregnant women using LMWH, it is suggested to discontinue LMWH every 12 to 24 hours before the planned delivery	98
	21. The use of elastic stockings associated with early mobility should be performed in all puerperal women	98

* Initially, this recommendation reached 75% agreement. After a deep discussion of the topic, 100% agreement was reached; ^†^ patients with laboratory tests positive for antiphospholipid syndrome and with a history of one miscarriage can be included in this group for treatment.

VTE: venous thromboembolism; AT: antitrombin III; FVL: factor V Leiden; IUD: intrauterine device; LMWH: low molecular weight heparin.

**As to screening**

**Investigate thrombophilia in women with a history of venous thromboembolism: 85% agreement**

Tests should be considered in patients in whom it is necessary to better understand the cause of the thrombotic event, in the evaluation of risk of recurrence, in the definition of anticoagulation time, and in the need for prophylaxis in situations of risk. Literature alerts us that tests should only be done when the results of these tests modify the management.^(1,6-8,11,12)^

**Investigate thrombophilia in first-degree kinship family members of patients with a history of venous thromboembolism, who potentially could be exposed to thrombogenic factors: 85% agreement**

Asymptomatic family members of patients with detected thrombophilia were recommended to be investigated, in order to avoid the association of thrombogenic factors, and to plan primary prophylaxis in situations of risk *e.g.* long trips, large operations). However, even in people with negative test results, this care should be considered.^(1,6-8,11,12)^

**Thrombophilia should be investigated in women with recurrent miscarriages: 88% agreement**

According to the American Society of Reproductive Medicine (ASRM), habitual or recurrent miscarriage is defined as the spontaneous and consecutive loss of two or more gestations before 20 to 22 weeks of gestational age. The difficulty of waiting for the occurrence of two miscarriages to perform an investigation of thrombophilia was and exposed and discussed; therefore, the consensus defined that investigation of thrombophilia tests can be provided to women with a history of only one gestational loss, but it is not mandatory.^(6,13)^

**Do not research thrombophilia routinely in all pregnant women during the prenatal care period: 99% agreement**

The presence of tests positive for thrombophilia in women with no personal or familial history does not necessarily mean an increased risk of thrombosis, generating medical care that is not standardized in literature. Cost-effectiveness studies have shown that the indiscriminate use of these tests was not effective in preventing deaths or other events.^(1,6-8,11,12)^ It was defined, therefore, that the investigation of thrombophilia is not routinely recommended for all pregnant women.

**Do not perform pre-contraception or pre-hormone replacement screening in the female population: 96% agreement**

In women at child-bearing age, the incidence of VTE in users of estrogen-progesterone (30 to 40 events per 100,000 people/year) and the estimated mortality due to VTE (3 per 1 million estrogen-progesterone users, and 14 per 1 million in estrogen-progesterone users with FVL mutation) are so low, that the number of women who would have to be tested in the laboratory for thrombophilia to prevent a death is very high (more than 92,000 with FVL). Therefore, there is no benefit in screening before the use of contraception or hormone replacement.^(1,6-9,11)^

**Perform screening before the use of hormones in women with history of thrombosis or with a positive familial history (first-degree kinship family members): 100% agreement**

The identification of hereditary thrombophilia in women with past history or family history of thrombosis aims to avoid the use of hormones in this population or minimize the risk with the use of hormones with a lower thrombogenic potential. However, it is important to point out that even the population that presents with negative screening may have a greater risk of VTE than the general population, because of presenting a positive family history.^(6-8,14,15)^

**As to time of the investigation**

**Avoid performing laboratory investigation of venous thromboembolism during the acute phase of thrombosis, since there could be a reduction in the levels of protein C, S, and antitrombin: 90% agreement**

The levels of protein C, S, antitrombin (AT) can be lower in the acute phase of thrombosis, which not always represents a true deficiency. Other level that should not be checked in this phase is lupus anticoagulant test, which can be influenced by the therapeutic anticoagulant. Another important data is that, during pregnancy and when using contraception, there may be a false protein S deficiency.^(6,7,16)^

Therefore, one should avoid investigating VTE during the acute phase of thrombosis.

**The best time to perform laboratory investigation of venous thromboembolism is at the end of anticoagulation, as of 2 to 4 weeks after discontinuing oral anticoagulation: 94% agreement**

The ideal time for laboratory investigation is controversial in literature. We will follow the most recommendation described in literature that perform 4 weeks after the end of anticoagulant therap.^(6,12,17)^

**Genetic tests for factor V Leiden and test of mutation of prothrombin, anticardiolipin, and anti-beta2-glycoprotein I do not suffer interference during the acute phase of thrombosis: 88% agreement**

The dosages of genetic factors (FVL and mutation of prothrombin) or titers of antibodies (anticardiolipin and beta2-glycoprotein) can be done at any time, since they do not suffer interference of the acute phase of thrombosis or from the use of anticoagulants.^(8,12,17)^

**As to the use of hormones**

**Women with a past history of thrombosis related to hormones present with a contraindication for use of hormones, except for progesterone intrauterine device: 95% agreement**

Despite the fact that this question had a high degree of agreement, it was questioned if there would be a difference in the incidence of thrombotic events relative to the route of administration and type of hormone.

After ample discussion and study,^(18-26)^ it was defined that:

- For women with an indication for contraception, the recommendation is for the use of a progesterone intrauterine device (IUD), which has proved to be safe in patients with history of thrombosis.^(20)^

- For the group of women in post-menopause, with an indication for hormone therapy and history of thrombosis, or who suffer from thrombophilia, there are studies that support the use of transdermal natural estrogens, due to the fact they have a lower risk as compared to oral agents, but there is no consensus in literature that consolidates this management.^(20,22)^

**Women with laboratory modifications of thrombophilia and with no past history or family history of thrombosis can use hormones with caution: 86% agreement**

The literature does not recommend an indiscriminate screening for investigation of thrombophilia in patients with no past or family history of thrombosis.^(1,7,8)^ However, since we still receive these patients with positive laboratory tests that were erroneously investigated, we defined hormones may be used with caution, balancing the risks and benefits, case by case, with decision made together by physician and patient, due to the difficulty of disregarding these positive results.

**Use of the levonorgestrel intrauterine device is safe in patients with thrombophilia: 100% agreement**

There is no association between the risk of VTE and the use of a progesterone IUD.^(7,27)^

**As to pregnancy**

**Women with previous venous thromboembolism associated with a transient risk and not related to pregnancy/contraception, should maintain surveillance during pregnancy; and during puerperium, they should perform antithrombotic prophylaxis for 6 weeks: 95% agreement**

Women who present with past history of thrombosis associated with a transient risk for VTE, such as long trips, postoperative periods etc., have the benefit of undergoing antithrombotic prophylaxis during the puerperium period.^(6)^

**Women with a previous venous thromboembolism associated with pregnancy/contraception should perform antithrombotic prophylaxis during pregnancy and in the puerperium: 98% agreement**

Pregnant women with a history of VTE present with the benefit of using antithrombotic prophylaxis during the entire gestation and puerperium periods, thus reducing the risk of VTE recurrence.^(6)^

**Women with factor V Leiden thrombophilia in homozygosis or mutant prothrombin, with no past venous thromboembolism and no family history of thrombosis should have prophylaxis only in the postpartum period: 75% agreement**

The literature recommends performing prophylaxis during puerperium (Level of Evidence 2B);^(6)^ however, due to great discordance during the meeting, it was established that there is need for prophylaxis both during pregnancy and puerperium. The rationale is in accordance with the new publication of October 2017, in which a systematic review of 36 meta-analyses demonstrated the due use of prophylaxis both in pregnancy and in the puerperium, regardless of women’s family history.^(28)^

Therefore, women with factor V Leiden thrombophilia in homozygosis or mutant prothrombin in homozygosis, with no previous VTE and no family history of thrombosis should have prophylaxis during pregnancy and puerperium.

**Women with factor V Leiden thrombophilia in homozygosis or mutant prothrombin in homozygosis, with no previous venous thromboembolism and with a family history of thrombosis, should have antithrombotic prophylaxis both during pregnancy and puerperium: 98% agreement**

The homozygous women for the mutation of the FVL and with a positive family history present with a chance lower those 47 events for each 1,000 when prophylaxis is used.^(6)^

**Women with thrombophilia (all other thrombophilias, except homozygous factor V Leiden and mutation of prothrombin 20210 homozygous), with no past thromboembolism, and with no family history of thrombosis should perform clinical vigilance during pregnancy and during puerperium: 92% agreement**

There are no data confirming efficacy of antithrombotic prophylaxis in this population, and clinical observation in these cases is recommended.^(6)^

**Women with thrombophilia (all other thrombophilias, except homozygous factor V Leiden and mutation of prothrombin 20210), with no previous venous thromboembolism event, and with family history of thrombosis, should perform clinical surveillance during pregnancy, and antithrombotic prophylaxis in the puerperium: 94% agreement**

In women with a positive family history for VTE and patients with a deficiency of antitrombin, of protein C or of protein S, antithrombotic prophylaxis demonstrates reduce the estimated number of VTE.^(4,6,29)^

**Women with antiphospholipid syndrome should use antithrombotic prophylaxis both during pregnancy and puerperium, combined with a low dose of acetylsalicylic acid (75-100mg/day): 98% agreement**

The investigation of antiphospholipid syndrome should be done in women with a history of arterial/VTE and obstetric morbidity (more than three consecutive early pregnancy losses, fetal death within or beyond 10 weeks of pregnancy and severe pre-eclampsia or placental insufficiency that need to deliver before 34 weeks of gestation). The laboratory criteria include persistent positivity for at least one test among lupus anticoagulant, anticardiolipin, and antibodies anti-beta-2-glycoprotein I, in which the laboratory tests should be performed with 12-week intervals. The clinical management of the pregnant patients with antiphospholipid syndrome aims to prevent obstetric complications and maternal thrombotic events.^(28)^ Combined therapy of a low dose of aspirin and heparin is considered the conventional treatment for patients with an established diagnosis of obstetric antiphospholipid syndrome, resulting in more than 70% of successful pregnancies.

On the other hand, the risk of VTE in pregnant women with no history of thrombosis and with positive antibody antiphospholipid is similar to the risk of the pregnant women with no antibodies, and there are no reasons for antithrombotic prophylaxis in this group of women.^(29)^

**In pregnant women on low molecular weight heparin, we recommend to discontinue it 12 to 24 hours before the planned delivery: 98% agreement**

Studies showed safety of performing an anesthetic block 12 hours after the last application of low molecular weight heparin at the prophylactic dose, and 24 hours after the last therapeutic dose of the medication, since the elimination half-life of the drug is 3 to 7 hours.^(6,30)^

**Elastic stockings associated with early mobility should be recommended to all puerperal women: 98% agreement**

The use of mechanical measures for the pregnant women hospitalized at the time of delivery (elastic stockings and intermittent pneumatic compression) and during the puerperium (elastic stockings) is recommended.^(6)^

## CONCLUSION

Over the last years, the recommendation of investigation and management for primary or secondary prevention of thrombosis in women with a past and family history has been evolving. However, laboratory thrombophilia tests are still used more frequently than the literature suggests, generating unnecessary costs and anxiety.

Hormone replacement therapy or the use of hormonal contraceptives, both in women with no risk and in those with history or risk of thrombophilia, should be individualized, with an adequate selection by means of a careful medical history taking, by appropriate administration route and choice of hormones.

In pregnant women, there is clinical management recommended and well-defined based on literature, which guides the prophylactic and therapeutic orientations during pregnancy and puerperium. Whereas in cases of non-pregnant women, in which studies are scarce and the variables are many, this consensus was based on guidelines, studies, and the opinion of experts to define a standard care for patient.

Thus, interdisciplinary guidelines for the investigation and prevention of thromboembolism were established, collaborating to a better management of patients and minimizing risks. This is the first national consensus.

## References

[1] 1. Middeldorp S. Inherited thrombophilia: a double-edged sword. Hematology Am Soc Hematol Educ Program. 2016;2016(1):1-9. Review. 10.1182/asheducation-2016.1.1 PMC6142488 27913455

[2] 2. James AH, Jamison MG, Brancazio LR, Myers ER. Venous thromboembolism during pregnancy and the postpartum period: incidence, risk factors, and mortality. Am J Obstet Gynecol. 2006;194(5):1311-5. 10.1016/j.ajog.2005.11.008 16647915

[3] 3. Meng K, Hu X, Peng X, Zhang Z. Incidence of venous thromboembolism during pregnancy and the puerperium: a systematic review and meta-analysis. J Matern Fetal Neonatal Med. 2015;28(3):245-53. Review. 10.3109/14767058.2014.913130 24716782

[4] 4. Kourlaba G, Relakis J, Kontodimas S, Holm MV, Maniadakis N. A systematic review and meta-analysis of the epidemiology and burden of venous thromboembolism among pregnant women. Int J Gynaecol Obstet. 2016;132(1):4-10. Review. 10.1016/j.ijgo.2015.06.054 26489486

[5] 5. Heit JA. Epidemiology of venous thromboembolism. Nat Rev Cardiol. 2015; 12(8):464-74. Review. 10.1038/nrcardio.2015.83 PMC4624298 26076949

[6] 6. Bates SM, Greer IA, Middeldorp S, Veenstra DL, Prabulos AM, Vandvik PO. VTE, thrombophilia, antithrombotic therapy, and pregnancy: Antithrombotic Therapy and Prevention of Thrombosis, 9th ed: American College of Chest Physicians Evidence-Based Clinical Practice Guidelines. Chest. 2012;141(2 Suppl):e691S-736S. 10.1378/chest.11-2300 PMC3278054 22315276

[7] 7. Moll S. Thrombophilia: clinical-practical aspects. J Thromb Thrombolysis. 2015;39(3):367-78. Review. 10.1007/s11239-015-1197-3 25724822

[8] 8. Stevens SM, Woller SC, Bauer KA, Kasthuri R, Cushman M, Streiff M, et al. Guidance for the evaluation and treatment of hereditary and acquired thrombophilia. J Thromb Thrombolysis. 2016;41(1):154-64. Review. 10.1007/s11239-015-1316-1 PMC4715840 26780744

[9] 9. Wu O, Greer IA. Is screening for thrombophilia cost-effective? Curr Opin Hematol. 2007;14(5):500-3. Review. 10.1097/MOH.0b013e32825f5318 17934357

[10] 10. Connors JM. Thrombophilia Testing and Venous Thrombosis. N Engl J Med. 2017;377(12):1177-87. Review. 10.1056/NEJMra1700365 28930509

[11] 11. Hicks LK, Bering H, Carson KR, Haynes AE, Kleinerman J, Kukreti V, et al. Five hematologic tests and treatments to question. Blood. 2014;124(24):3524-8. 10.1182/blood-2014-09-599399 25472968

[12] 12. Baglin T, Gray E, Greaves M, Hunt BJ, Keeling D, Machin S, Mackie I, Makris M, Nokes T, Perry D, Tait RC, Walker I, Watson H; British Committee for Standards in Haematology. Clinical guidelines for testing for heritable thrombophilia. Br J Haematol. 2010;149(2):209-20. 10.1111/j.1365-2141.2009.08022.x 20128794

[13] 13. Practice Committee of the American Society for Reproductive Medicine. Definitions of infertility and recurrent pregnancy loss. Fertil Steril. 2008;89(6):1603. 10.1016/j.fertnstert.2008.03.002 18485348

[14] 14. Ray JG, Chan WS. Deep vein thrombosis during pregnancy and the puerperium: a meta-analysis of the period of risk and the leg of presentation. Obstet Gynecol Surv. 1999;54(4):265-71. 10.1097/00006254-199904000-00023 10198931

[15] 15. Sørensen HT, Riis AH, Diaz LJ, Andersen EW, Baron JA, Andersen PK. Familial risk of venous thromboembolism: a nationwide cohort study. J Thromb Haemost. 2011;9(2):320-4. 10.1111/j.1538-7836.2010.04129.x 21040446

[16] 16. Kurasawa G, Kotani K, Ito Y, Saiga K, Iijima K. Reduction in protein S activity during normal pregnancy. Aust N Z J Obstet Gynaecol. 2007;47(3):213-5. 10.1111/j.1479-828X.2007.00720.x 17550488

[17] 17. Jennings I, Kitchen S, Woods TA, Preston FE. Multilaboratory testing in thrombophilia through the United Kingdom National External Quality Assessment Scheme (Blood Coagulation) Quality Assurance Program. Semin Thromb Hemost. 2005;31(1):66-72. 10.1055/s-2005-863807 15706477

[18] 18. van Hylckama Vlieg A, Helmerhorst FM, Vandenbroucke JP, Doggen CJ, Rosendaal FR. The venous thrombotic risk of oral contraceptives, effects of oestrogen dose and progestogen type: results of the MEGA case-control study. BMJ. 2009;339:b2921. 10.1136/bmj.b2921 PMC2726929 19679614

[19] 19. Lidegaard Ø, Nielsen LH, Skovlund CW, Skjeldestad FE, Løkkegaard E. Risk of venous thromboembolism from use of oral contraceptives containing different progestogens and oestrogen doses: danish cohort study, 2001-9. BMJ. 2011;343:d6423. 10.1136/bmj.d6423 PMC3202015 22027398

[20] 20. Canonico M, Oger E, Plu-Bureau G, Conard J, Meyer G, Lévesque H, Trillot N, Barrellier MT, Wahl D, Emmerich J, Scarabin PY; Estrogen and Thromboembolism Risk (ESTHER) Study Group. Hormone therapy and venous thromboembolism among postmenopausal women: impact of the route of estrogen administration and progestogens: the ESTHER study. Circulation. 2007;115(7):840-5. 10.1161/CIRCULATIONAHA.106.642280 17309934

[21] 21. Lidegaard Ø, Løkkegaard E, Svendsen AL, Agger C. Hormonal contraception and risk of venous thromboembolism: national follow-up study. BMJ. 2009; 339:b2890. 10.1136/bmj.b2890 PMC2726928 19679613

[22] 22. de Bastos M, Stegeman BH, Rosendaal FR, Van Hylckama Vlieg A, Helmerhorst FM, Stijnen T, et al. Combined oral contraceptives: venous thrombosis. Cochrane Database Syst Rev. 2014;(3):CD010813. Review. 10.1002/14651858.CD010813.pub2 PMC10637279 24590565

[23] 23. van Hylckama Vlieg A, Middeldorp S. Hormone therapies and venous thromboembolism: where are we now? J Thromb Haemost. 2011;9(2):257-66. Review. 10.1111/j.1538-7836.2010.04148.x 21114755

[24] 24. L’Hermite M. HRT optimization, using transdermal estradiol plus micronized progesterone, a safer HRT. Climacteric. 2013;16(Suppl 1):44-53. Review. 10.3109/13697137.2013.808563 23848491

[25] 25. Vieira CS, Ferriani RA, Garcia AA, Pintão MC, Azevedo GD, Gomes MK, et al. Use of the etonogestrel-releasing implant is associated with hypoactivation of the coagulation cascade. Hum Reprod. 2007;22(8):2196-201. 10.1093/humrep/dem153 17569674

[26] 26. Roumen FJ, Mishell DR Jr. The contraceptive vaginal ring, NuvaRing^(®)^, a decade after its introduction. Eur J Contracept Reprod Health Care. 2012;17(6):415-27. Review. 10.3109/13625187.2012.713535 23113828

[27] 27. van Hylckama Vlieg A, Helmerhorst FM, Rosendaal FR. The risk of deep venous thrombosis associated with injectable depot-medroxyprogesterone acetate contraceptives or a levonorgestrel intrauterine device. Arterioscler Thromb Vasc Biol. 2010;30(11):2297-300. 10.1161/ATVBAHA.110.211482 20798377

[28] 28. Croles FN, Nasserinejad K, Duvekot JJ, Kruip MJ, Meijer K, Leebeek FW. Pregnancy, thrombophilia, and the risk of a first venous thrombosis: systematic review and bayesian meta-analysis. BMJ. 2017;359:j4452. Review. 10.1136/bmj.j4452 PMC5657463 29074563

[29] 29. Santos TD, Ieque AL, de Carvalho HC, Sell AM, Lonardoni MV, Demarchi IG, et al. Antiphospholipid syndrome and recurrent miscarriage: A systematic review and meta-analysis. J Reprod Immunol. 2017;123:78-87. Review. 10.1016/j.jri.2017.09.007 28985591

[30] 30. Working Party; Association of Anaesthetists of Great Britain & Ireland; Obstetric Anaesthetists’ Association; Regional Anaesthesia UK. Regional anaesthesia and patients with abnormalities of coagulation: the Association of Anaesthetists of Great Britain & Ireland The Obstetric Anaesthetists’ Association Regional Anaesthesia UK. Anaesthesia. 2013;68(9):966-72. Erratum in: Anaesthesia. 2016;71(3):352. 10.1111/anae.12359 23905877

