# Supplementary material for: Consensus on the investigation of thrombophilia in women and clinical management
Source: Einstein (Sao Paulo). 2019 Aug 13;17(3):eAE4510. doi: 10.31744/einstein_journal/2019AE4510 (PMC6707753; doi:10.31744/einstein_journal/2019AE4510)
Supplement: Translation

# Translation: Consenso sobre a investigação de trombofilia em mulheres e manejo clínico

**Authors:** Claudia Mac Donald Bley Nascimento, Andréa Maria Novaes Machado, João Carlos de Campos Guerra, Eduardo Zlotnik, Dirceu Hamilton Cordeiro Campêlo, Paulo Kauffman, Hilton Waksman, Nelson Wolosker, Sérgio Podgaec, Nelson Hamerschlak

**Affiliations:** 1 Hospital Israelita Albert Einstein, São Paulo, SP, Brasil.; 2 Hospital das Clínicas, Faculdade de Medicina, Universidade de São Paulo, São Paulo, SP, Brasil.

**Keywords:** Trombofilia, Síndrome antifosfolipídica, Tromboembolia venosa, Gravidez, Aborto habitual, Complicações na gravidez, Anticoncepção, Trombose venosa

## Abstract

**Objetivo:**

Padronizar a investigação e o manejo clínico de mulheres com anormalidades clínicas e exames laboratoriais sugestivos de trombofilia, para melhorar a abordagem antitrombótica e otimizar a indicação de exames laboratoriais.

**Metodologia:**

Foi conduzida discussão incluindo 107 médicos (ginecologistas/obstetras, hematologistas e cirurgiões vasculares) participantes de um fórum realizado no Hospital Israelita Albert Einstein, em São Paulo (SP). Como critério mínimo, estabeleceu-se concordância de 80% em votação para cada recomendação de conduta registrada em documento como diretrizes finais. Os casos em que a concordância esteve abaixo de 80% foram rediscutidos, para definir consenso na conduta.

**Conclusão:**

A padronização e o estabelecimento de consenso institucional, com sugestões para abordagem clínica, contribui para melhorar o manejo do grupo a ser avaliado e minimizar os riscos de intercorrências. Este foi o primeiro consenso nacional sobre investigação de trombofilia em mulheres.

## INTRODUÇÃO

A trombofilia é definida como tendência à trombose decorrente de alterações hereditárias (deficiência de proteína C, S e antitrombina; mutação do fator V de Leiden − FVL; e mutação do gene da protrombina) ou adquiridas da coagulação (síndrome antifosfolípide), que levam a estado pró-trombótico, o qual predispõe a tromboses venosas ou arteriais. A etiologia da trombose (oclusão arterial ou venosa do vaso sanguíneo por um coágulo) é multifatorial, e a presença de uma alteração laboratorial genética ou adquirida de trombofilia é apenas um dos muitos fatores que determinam seu risco, tendo pouca prevalência na população geral, conforme demonstrado na tabela 1. Obesidade, uso de hormônios, cirurgia, longos períodos no leito, viagem de avião de longa distância, câncer, tabagismo e gravidez são fatores que aumentam o risco trombótico de forma independente.^(1)^

**Tabela 1:** Prevalência de trombofilia e risco relativo estimado para diversas manifestações clínicas(1)

	Deficiência de antitrombina	Deficiência da proteína C	Deficiência da proteína S	Fator V de Leiden	Mutação do gene da protrombina (20210A)
Prevalência na população geral, %	0,02	0,2	0,03-0,13	3,0-7,0	0,7-4,0
Prevalência nos pacientes com TEV recorrente, %	1,0	3,0	2,0	20,0	5,0
Risco relativo para primeira manifestação de TEV	5-10	4-6,5	1-10	3-5	2-3
Risco relativo para recorrência de TEV	1,9-2,6	1,4-1,8	1,0-1,4	1,4	1,4
Risco relativo para trombose arterial	Sem associação	Sem associação consistente	Sem associação consistente	1,3	0,9
Risco relativo para complicações obstétricas	1,3-3,6	1,3-3,6	1,3-3,6	1,0-2,6	0,9-1,3

TEV: tromboembolismo venoso.

A gravidez está associada a importantes alterações fisiológicas, que afetam a coagulação e o sistema fibrinolítico. Um desequilíbrio neste sistema leva a um estado de hipercoagulabilidade, e as mulheres grávidas têm, desse modo, risco aumentado de eventos de tromboembolismo venoso (TEV).^(2)^ A incidência de tromboembolismo em mulheres grávidas é de 0,6 a 1,7 caso em mil gestações.^(3)^

Cerca de 50 a 60% dessas tromboses ocorrem durante o puerpério (até 6 semanas pós-parto).^(4)^

Apesar de o risco de TEV ser cerca de quatro vezes maior na gravidez do que nas mulheres não grávidas em idade fértil,^(5)^ não há evidência científica de que a incorporação da pesquisa de trombofilia no pré-natal, como muitas vezes é solicitada, seja útil.

A literatura preconiza que a investigação laboratorial de trombofilia seja norteada por anamnese e histórico familiar de tromboses, sendo preconizada quando seu resultado impactará potencialmente na conduta clínica. A investigação deve ser realizada nas seguintes situações:^(1,6-8)^

- Pacientes com histórico de tromboses e seus familiares de primeiro grau, caso o conhecimento de resultados dos testes laboratoriais modifique a conduta médica.

- Pacientes com história de abortamento tardio ou abortamentos precoces de repetição.

Estudos de custo-efetividade mostraram que o uso indiscriminado dos testes de trombofilia na população geral não foram efetivos em prevenir mortes ou outros eventos.^(9-11)^

## OBJETIVO

Com o intuito de padronizar a investigação laboratorial de trombofilia na população feminina, decidiu-se, junto ao corpo clínico do Hospital Israelita Albert Einstein, criar o Fórum de Trombofilia, formado por hematologistas, cirurgiões vasculares e ginecologistas/obstetras da instituição, para avaliar a real importância desta investigação, sua conduta e definir, por consenso, a uniformização das medidas que devem ser tomadas no cuidado com nosso público-alvo.

O Fórum de Trombofilia foi realizado em 10 de junho de 2017, no Hospital Israelita Albert Einstein, em São Paulo (SP).

## METODOLOGIA

A equipe de hematologia do Hospital Israelita Albert Einstein (HIAE) realizou uma busca, em abril de 2017, de artigos científicos publicados no PubMed^®^/MEDLINE^®^ entre janeiro de 2007 e janeiro de 2017, inserindo as palavras em inglês: (“*thrombophilia*”), (“*thrombosis and pregnancy*”), (“*thrombophilia and screening*”) e (“*pre-hormone therapy*”). Os artigos recuperados foram analisados quanto a relevância, escopo e desenho de estudo, gerando uma lista de estudos clínicos randomizados, metanálises, revisões sistemáticas e diretrizes,^(1,6-11)^ que foi encaminhada como sugestão de leitura via *e-mail* ao corpo clínico dos 539 médicos especialistas cadastrados em hematologia, cirurgia vascular, e ginecologia e obstetrícia, junto de uma carta-convite para o fórum, agendado para 2 semanas depois do recebimento deste *e-mail*. Destes, 107 médicos (80% ginecologistas/obstetras, 10% hematologistas e 10% cirurgiões vasculares) compareceram ao fórum, que teve duração de 4 horas e 30 minutos, sendo composto por três etapas: aula teórica para exposição do tema trombofilia em mulheres; apresentação interativa de casos clínicos, na qual os participantes do fórum podiam expor seus questionamentos e comentários; e exposição de questionário elaborado pela equipe de hematologia do HIAE, com 21 assertivas que ilustravam as principais condutas médicas sobre o tema abordado (trombofilia em mulheres). O público presente deveria avaliar cada assertiva e votar como verdadeiro, para concordar com a conduta médica explanada, ou falso, para discordar dela. Um sistema eletrônico de votação foi instalado no auditório, com equipamentos individuais, permitindo o registro do percentual de respostas (verdadeiro e falso).

Foi estabelecida, pelo grupo de médicos presentes no fórum, como critério mínimo de resolução aceitável, a concordância de 80% na votação para inserção de cada recomendação, sendo então a assertiva inserida no documento final. Os casos em que a concordância ficasse abaixo de 80% seriam rediscutidos, chegando-se a um acordo consensual de conduta majoritária.

## RECOMENDAÇÕES

Após revisão da literatura, leitura da bibliografia, exposição da literatura em sessão plenária, discussão dinâmica de casos clínicos e votação do consenso, obtivemos concordância acima de 80% em 95% das questões (20 das 21), e somente uma das questões obteve votação menor que 80%. Esta questão, após ampla discussão, foi reformulada e acordada por unanimidade.

As diretrizes estabelecidas estão resumidas na tabela 2.

**Tabela 2:** Recomendações gerais e percentual de concordância deste consenso de trombofilia

	Recomendações gerais	Concordância (%)
***Screening***	1. Investigar trombofilia em mulheres com histórico de TEV	85
	2. Investigar trombofilia em parentes de primeiro grau de pacientes com histórico de TEV e que potencialmente possam ser expostos a fatores trombogênicos	85
	3. Investigar trombofilia em mulheres com abortos recorrentes	88
	4. Não se recomenda pesquisar trombofilia rotineiramente em todas as gestantes no pré-natal	99
	5. Não se recomenda realizar *screening* pré-anticoncepcional ou pré-reposição hormonal na população feminina	96
	6. Realizar *screening* pré-uso de hormônios em mulheres com antecedente de tromboses ou com história familiar positiva (familiares de primeiro grau)	100
**Momento da investigação**	7. Evitar realizar investigação laboratorial de TEV na fase aguda da trombose, pois pode ocorrer redução dos níveis plasmáticos de proteína C, S e AT	90
	8. O melhor momento para realizar a investigação laboratorial de TEV é ao final do término da anticoagulação, a partir de 2-4 semanas após a suspensão da anticoagulação	94
	9. Testes genéticos para FVL e pesquisa da mutação da protrombina, anticardiolipina e anti-B2-glicoproteína I não sofrem interferência da fase aguda da trombose	88
**Uso de hormônios**	10. Mulheres com histórico prévio de trombose relacionada a hormônios apresentam contraindicação ao uso de hormônios, exceto DIU de progesterona	95
	11. Mulheres com alteração laboratorial de trombofilia e sem histórico de tromboses podem usar hormônios com cautela	86
	12. O uso de DIU com levonorgestrel é seguro nas pacientes com trombofilia	100
**Gestação e puerpério**	13. Mulheres com TEV prévio, associado a risco transitório e sem relação com gravidez/contracepção, devem manter vigilância durante a gestação e, durante o puerpério, devem realizar profilaxia antitrombótica por 6 semanas	95
	14. Mulheres com TEV prévio associado a gravidez/contracepção devem realizar profilaxia antitrombótica durante a gestação e no puerpério	98
	15. Mulheres com trombofilia FVL em homozigose ou protrombina mutante em homozigose, sem TEV prévio e sem histórico familiar de trombose, devem realizar profilaxia durante a gestação e no puerpério	100*
	16. Mulheres com trombofilia FVL em homozigose ou protrombina mutante em homozigose, sem TEV prévio e com histórico familiar de trombose, devem realizar profilaxia antitrombótica somente no pós-parto	98
	17. Mulheres com trombofilia (todas as outras trombofilias, exceto homozigotas FVL e mutação da protrombina 20210 homozigotas), sem tromboembolismo prévio e sem histórico familiar de trombose, devem realizar vigilância clínica no período anteparto e no puerpério	92
	18. Mulheres com trombofilia (todas as outras trombofilias, exceto homozigotas FVL e mutação da protrombina 20210), sem tromboembolismo prévio e com histórico familiar de trombose devem realizar vigilância clínica durante a gestação e profilaxia antitrombótica no puerpério	94
	19. Mulheres com síndrome antifosfolipídeo devem fazer uso de profilaxia antitrombótica tanto no período anteparto quanto no puerpério, combinada com baixa dose de aspirina (75-100mg/dia)^†^	98
	20. Nas mulheres grávidas em uso de HBPM, recomenda-se descontinuar a HBPM 12 a 24 horas antes de parto planejado	98
	21. O uso de meias elásticas associado a mobilidade precoce deve ser realizado em todas as mulheres puérperas	98

* Inicialmente, esta recomendação atingiu 75% de concordância. Após discussão aprofundada do tema, obteve-se 100% de concordância; ^†^ pacientes com testes laboratoriais positivos para síndrome antifosfolipídeo e histórico de um abortamento podem ser incluídas neste grupo para tratamento.

TEV: tromboembolismo venoso; AT: antitrombina; FVL: fator V de Leiden; DIU: dispositivo intrauterino; HBPM: heparina de baixo peso molecular.

**Em relação ao *screening***

**Investigar trombofilia em mulheres com histórico de tromboembolismo venoso: concordância de 85%**

Testes devem ser considerados em pacientes nos quais é necessário entender melhor a causa do evento trombótico, a avaliação de risco de recorrência, a definição do tempo de anticoagulação e a necessidade de profilaxia em situações de risco. A literatura alerta que devem ser realizados testes somente quando os resultados desses exames modificam a conduta.^(1,6-8,11,12)^

**Investigar trombofilia em parentes de primeiro grau de pacientes com histórico de tromboembolismo venoso e que potencialmente possam ser expostos a fatores trombogênicos: concordância de 85%**

Foi recomendado realizar investigação em familiares assintomáticos de pacientes com trombofilia detectada, a fim de evitar associação de fatores trombogênicos e planejar a profilaxia primária em situações de risco (por exemplo: viagens longas e grandes cirurgias). Porém, mesmo em pessoas com exames negativos, esse cuidado deve ser considerado.^(1,6-8,11,12)^

**Devemos investigar trombofilia em mulheres com abortos recorrentes: concordância de 88%**

De acordo com a *American Society of Reproductive Medicine* (ASRM), aborto habitual ou recorrente é definido como a perda espontânea e consecutiva de duas ou mais gestações antes de 20 a 22 semanas de idade gestacional. Foi exposta e discutida a dificuldade de aguardar a ocorrência de dois abortos para a realização da investigação de trombofilia; portanto, foi definido, no consenso, que se pode oferecer investigação de testes de trombofilia em mulheres com histórico de apenas uma perda gestacional, porém não é obrigatório.^(6,13)^

**Não pesquisar trombofilia rotineiramente em todas as gestantes no pré-natal: concordância de 99%**

A presença de testes positivos de trombofilia em mulheres sem histórico pessoal ou familiar não necessariamente significa aumento do risco de trombose, gerando conduta médica não padronizada na literatura. Estudos de custo-efetividade mostraram que o uso indiscriminado destes testes não foi efetivo em prevenir mortes ou outros eventos.^(1,6-8,11,12)^ Definiu-se, portanto, que a pesquisa de trombofilia não está recomendada de rotina para todas as gestantes.

**Não realizar *screening* pré-anticoncepcional ou pré-reposição hormonal na população feminina: concordância de 96%**

Em mulheres em idade reprodutiva, a incidência de TEV em usuárias de estrógeno-progesterona (30 a 40 eventos por 100 mil pessoas/ano) e a mortalidade estimada por TEV (3 por 1 milhão em usuárias de estrógeno-progesterona, e 14 por 1 milhão em usuárias de estrógeno-progesterona com mutação do FVL) é tão baixa, que o número de mulheres que teriam que ser testadas laboratorialmente para trombofilia para prevenir uma morte é muito alto (mais de 92 mil portadoras de FVL). Portanto, não há benefício em se realizar rastreamento antes do uso de contraceptivo ou reposição hormonal.^(1,6-9,11)^

**Realizar *screening* antes do uso de hormônios em mulheres com antecedente de tromboses ou com história familiar positiva (familiares de primeiro grau): concordância de 100%**

A identificação de trombofilia hereditária em mulheres com antecedentes pessoais ou histórico familiar de trombose visa evitar o uso de hormônio nesta população ou minimizar o risco com o uso de hormônios com potencial menos trombogênico. Porém é importante ressaltar que mesmo a população que apresenta *screening* negativo pode ter um risco maior de TEV que a população geral, pelo fato de apresentar história familiar positiva.^(6-8,14,15)^

**Em relação ao momento da investigação**

**Evitar realizar investigação laboratorial de tromboembolismo venoso na fase aguda da trombose, pois pode ocorrer redução dos níveis de proteína C, S e antitrombina III: concordância de 90%**

As dosagens da proteína C, S, antitrombina III (AT) podem mostrar-se mais baixas na fase aguda da trombose, mas nem sempre representando deficiência real. Outra dosagem que não deve ser realizada nesta fase é a pesquisa de anticoagulante lúpico, que pode sofrer influência do anticoagulante terapêutico. Outro dado importante é que, durante a gestação e em uso de contracepção, pode haver falsa deficiência de proteína S.^(6,7,16)^

Portanto, deve-se evitar investigar TEV na fase aguda da trombose.

**O melhor momento para realizar a investigação laboratorial de tromboembolismo venoso é ao final do término da anticoagulação, a partir de 2 a 4 semanas após a suspensão da anticoagulação oral: concordância de 94%**

O momento ideal de investigação laboratorial é controverso na literatura. Seguiremos a preconização de realizar após 4 semanas do término da terapia anticoagulante, por ser o mais descrito em literatura.^(6,12,17)^

**Testes genéticos para fator V de Leiden e pesquisa da mutação da protrombina, anticardiolipina e antibeta-2-glicoproteína I não sofrem interferência da fase aguda da trombose: concordância de 88%**

As dosagens de fatores genéticos (FVL e mutação da protrombina) ou títulos de anticorpos (anticardiolipinas e beta-2-glicoproteínas) podem ser realizados a qualquer momento, pois não sofrem interferência da fase aguda da trombose ou do uso de anticoagulante.^(8,12,17)^

**Em relação ao uso de hormônios**

**Mulheres com histórico prévio de trombose relacionada a hormônios apresentam contraindicação ao uso de hormônios, exceto dispositivo intrauterino de progesterona: concordância de 95%**

Apesar de esta questão ter tido alta concordância, foi questionado se haveria diferença na incidência de eventos trombóticos em relação à via de administração e ao tipo de hormônio.

Após ampla discussão e estudo,^(18-26)^ foi definido que:

- Para mulheres com indicação de contracepção, preconiza-se o uso de dispositivo intrauterino (DIU) de progesterona, que se mostra seguro em pacientes com antecedentes trombóticos.^(20)^

- Para o grupo de mulheres na pós-menopausa, com indicação de terapia hormonal e antecedentes trombóticos, ou portadoras de trombofilia, existem estudos que suportam o uso de estrogênios naturais e por via transdérmica, pelo fato de terem menor risco em relação ao uso oral, porém não há consenso na literatura que consolide esta conduta.^(20,22)^

**Mulheres com alteração laboratorial de trombofilia e sem histórico pessoal ou familiar de tromboses podem usar hormônios com cautela: concordância de 86%**

A literatura não preconiza a realização *screening* indiscriminado de investigação de trombofilia em pacientes sem histórico pessoal ou familiar de trombose.^(1,7,8)^ Porém, pelo fato de ainda recebermos essas pacientes com testes laboratoriais positivos erroneamente investigados, definimos que podem ser usado hormônios com cautela, ponderando riscos e benefícios caso a caso, com decisão conjunta médico-paciente, pela dificuldade de menosprezar tais resultados positivos.

**O uso de dispositivo intrauterino com levonorgestrel é seguro nas pacientes com trombofilia: concordância de 100%**

Não há associação entre risco de TEV e uso de DIU de progesterona.^(7,27)^

**Em relação à gestação**

**Mulheres com tromboembolismo venoso prévio, associado a risco transitório e sem relação com gravidez/contracepção, devem manter vigilância durante a gestação e, durante o puerpério, realizar profilaxia antitrombótica por 6 semanas: concordância de 95%**

Mulheres que apresentaram antecedente de trombose associado a risco transitório para TEV, como viagens de longa distância, pós-operatórios etc., têm benefício de realizar profilaxia antitrombótica no período do puerpério.^(6)^

**Mulheres com tromboembolismo venoso prévio associado à gravidez/contracepção devem realizar profilaxia antitrombótica durante a gestação e no puerpério: concordância de 98%**

Gestantes com histórico de TEV apresentam benefício do uso de profilaxia antitrombótica durante todo período gestacional e puerpério, reduzindo o risco de recorrência de TEV.^(6)^

**Mulheres com trombofilia fator V de Leiden em homozigose ou protrombina mutante, sem tromboembolismo venoso prévio e sem histórico familiar de trombose, devem realizar profilaxia somente no pós-parto: concordância de 75%**

A literatura preconiza realizar profilaxia no puerpério (Nível de Evidência 2B),^(6)^ porém, devido à grande discordância durante a reunião, ficou estabelecida a necessidade de profilaxia tanto na gestação quanto no puerpério. A justificativa está de acordo com nova publicação de outubro de 2017 de revisão sistemática de 36 metanálises que evidenciou uso devido de profilaxia tanto na gestação quanto no puerpério em mulheres independente de sua história familiar.^(28)^

Portanto, mulheres com trombofilia FVL em homozigose ou protrombina mutante em homozigose, sem TEV prévio e sem histórico familiar de trombose, devem realizar profilaxia durante a gestação e no puerpério.

**Mulheres com trombofilia fator V de Leiden em homozigose ou protrombina mutante em homozigose, sem tromboembolismo venoso prévio e com histórico familiar de trombose devem realizar profilaxia antitrombótica tanto durante a gestação quanto no puerpério: concordância de 98%**

As mulheres homozigóticas para a mutação do FVL e com história familiar positiva apresentam chance menor que 47 eventos a cada mil quando a profilaxia é utilizada.^(6)^

**Mulheres com trombofilia (todas as outras trombofilias, exceto homozigotas fator V de Leiden e mutação da protrombina 20210 homozigotas), sem tromboembolismo prévio e sem histórico familiar de trombose devem realizar vigilância clínica durante a gestação e no puerpério: concordância de 92%**

Não há dados que comprovem eficácia de uso de profilaxia antitrombótica nesta população, e a observação clínica, nesses casos, é preconizada.^(6)^

**Mulheres com trombofilia (todas as outras trombofilias, exceto homozigotas fator V de Leiden e mutação da protrombina 20210), sem tromboembolismo venoso prévio e com histórico familiar de trombose devem realizar vigilância clínica durante a gestação e profilaxia antitrombótica no puerpério: concordância de 94%**

Em mulheres com história familiar positiva para TEV e portadoras de deficiência de antitrombina III, de proteína C ou de proteína S, a profilaxia antitrombótica reduz o número estimado de TEV.^(4,6,29)^

**Mulheres com síndrome antifosfolipídeo devem fazer uso de profilaxia antitrombótica tanto durante a gestação quanto no puerpério, combinada com baixa dose de ácido acetilsalicílico (75 a 100mg/dia): concordância de 98%**

A pesquisa de síndrome antifosfolipídeo deve ser realizada em mulheres com história de trombose arterial/venosa e morbidade obstétrica (mais de três perdas gestacionais precoces consecutivas, morte fetal em ou além de 10 semanas de gestação, e pré-eclâmpsia grave ou insuficiência placentária que necessitam de parto antes de 34 semanas de gestação). Os critérios laboratoriais incluem a positividade persistente para pelo menos um teste entre anticoagulante lúpico, anticardiolipina e anticorpos antibeta-2-glicoproteína I, devendo os testes laboratoriais serem realizados com intervalos de 12 semanas. O manejo clínico de pacientes grávidas com síndrome antifosfolipídeo tem como objetivo prevenir complicações obstétricas e eventos trombóticos maternos.^(28)^ A terapia combinada de baixa dose de aspirina e heparina é considerada tratamento convencional para pacientes com diagnóstico estabelecido de síndrome antifosfolipídeo obstétrica, resultando em mais de 70% de gestações bem-sucedidas.

Já o risco de TEV em gestantes sem antecedentes de tromboses e com a presença de anticorpo antifosfolipídeo positivo é semelhante ao de gestante sem anticorpo presente, sem justificativa para profilaxia antitrombótica neste grupo de mulheres.^(29)^

**Nas mulheres grávidas em uso de heparina de baixo peso molecular, recomenda-se descontinuá-la 12 a 24 horas antes de parto planejado: concordância de 98%**

Estudos mostram segurança de realização de bloqueio anestésico 12 horas após a última aplicação de heparina de baixo peso molecular na dose profilática, e 24 horas após a última dose terapêutica da medicação, sendo a meia-vida de eliminação da droga de 3 a 7 horas.^(6,30)^

**Meias elásticas associadas à mobilidade precoce devem ser recomendadas a todas as puérperas: concordância de 98%**

Recomenda-se o uso de medidas mecânicas para as gestantes internadas por ocasião do parto (meias elásticas e compressão pneumática intermitente) e durante o puerpério (meias elásticas).^(6)^

## CONCLUSÃO

Nos últimos anos, a preconização de investigação e conduta de prevenção primária ou secundária de trombose nas mulheres com histórico pessoal e familiar vem evoluindo. Porém, ainda são realizados testes laboratoriais de trombofilia mais frequentemente do que a literatura preconiza, gerando custos e ansiedade desnecessários.

A terapia de reposição hormonal ou o uso de contraceptivos hormonais, tanto nas mulheres sem risco como nas que têm antecedentes ou risco de trombofilia, devem ser individualizados e com seleção adequada por meio de anamnese cuidadosa, via de administração apropriada e escolha dos hormônios.

Em gestantes, há condutas preconizadas e bem definidas, com base na literatura, que norteiam as orientações profiláticas e terapêuticas durante a gestação e o puerpério. Já nos casos das mulheres não grávidas, em que os estudos são escassos e as variáveis são muitas, este consenso se baseou em diretrizes, estudos e opinião de *experts* para definir uma padronização de conduta do atendimento.

Assim, foram estabelecidas diretrizes de forma interdisciplinar, para investigação e prevenção de tromboembolismo, colaborando com um melhor manejo dos pacientes e minimizando riscos. Este é o primeiro consenso nacional.
